# Glycolysis: An early marker for vancomycin‐specific T‐cell activation

**DOI:** 10.1111/cea.14423

**Published:** 2024-01-04

**Authors:** Joshua Gardner, Sean Hammond, Rebecca Jensen, Andrew Gibson, Matthew S. Krantz, Michael Ardern‐Jones, Elizabeth J. Phillips, Munir Pirmohamed, Amy E. Chadwick, Catherine Betts, Dean J. Naisbitt

**Affiliations:** ^1^ Department of Pharmacology and Therapeutics, Centre for Drug Safety Science University of Liverpool Liverpool UK; ^2^ ApconiX Alderley Edge UK; ^3^ Murdoch University Institute for Immunology & Infectious Diseases Perth Western Australia Australia; ^4^ Vanderbilt Institute for Infection, Immunology and Inflammation Vanderbilt University Nashville Tennessee USA; ^5^ Clinical Experimental Sciences University of Southampton Faculty of Medicine, Sir Henry Wellcome Laboratories, Southampton General Hospital Southampton UK; ^6^ Clinical Pharmacology & Safety Sciences AstraZeneca R&D Cambridge UK

**Keywords:** DRESS, drug hypersensitivity, glycolysis, T lymphocytes, vancomycin

## Abstract

**Background:**

Vancomycin, a glycopeptide antibiotic used for Gram‐positive bacterial infections, has been linked with drug reaction with eosinophilia and systemic symptoms (DRESS) in HLA‐A*32:01‐expressing individuals. This is associated with activation of T lymphocytes, for which glycolysis has been isolated as a fuel pathway following antigenic stimulation. However, the metabolic processes that underpin drug‐reactive T‐cell activation are currently undefined and may shed light on the energetic conditions needed for the elicitation of drug hypersensitivity or tolerogenic pathways. Here, we sought to characterise the immunological and metabolic pathways involved in drug‐specific T‐cell activation within the context of DRESS pathogenesis using vancomycin as model compound and drug‐reactive T‐cell clones (TCCs) generated from healthy donors and vancomycin‐hypersensitive patients.

**Methods:**

CD4+ and CD8+ vancomycin‐responsive TCCs were generated by serial dilution. The Seahorse XFe^96^ Analyzer was used to measure the extracellular acidification rate (ECAR) as an indicator of glycolytic function. Additionally, T‐cell proliferation and cytokine release (IFN‐γ) assay were utilised to correlate the bioenergetic characteristics of T‐cell activation with in vitro assays.

**Results:**

Model T‐cell stimulants induced non‐specific T‐cell activation, characterised by immediate augmentation of ECAR and rate of ATP production (JATPglyc). There was a dose‐dependent and drug‐specific glycolytic shift when vancomycin‐reactive TCCs were exposed to the drug. Vancomycin‐reactive TCCs did not exhibit T‐cell cross‐reactivity with structurally similar compounds within proliferative and cytokine readouts. However, cross‐reactivity was observed when analysing energetic responses; TCCs with prior specificity for vancomycin were also found to exhibit glycolytic switching after exposure to teicoplanin. Glycolytic activation of TCC was HLA restricted, as exposure to HLA blockade attenuated the glycolytic induction.

**Conclusion:**

These studies describe the glycolytic shift of CD4+ and CD8+ T cells following vancomycin exposure. Since similar glycolytic switching is observed with teicoplanin, which did not activate T cells, it is possible the master switch for T‐cell activation is located upstream of metabolic signalling.

## Graphical abstract

Vancomycin has been linked with drug reaction with DRESS in HLA‐A*32:01 expressing individuals and is associated with activation of drug‐specific T lymphocytes. However, the metabolic processes than underpin drug‐reactive T‐cell activation are undefined. CD4+ and CD8+ vancomycin‐specific T‐cell clones (TCCs) generated from healthy donors and hypersensitive patients expressing HLA‐A*32:01 undergo dose‐dependent, drug‐specific and HLA restricted glycolytic switching when activated with vancomycin. TCCs displayed ‘energetic’ cross‐reactivity with teicoplanin, indicating the master switch for T‐cell activation may be located upstream of metabolic signalling.

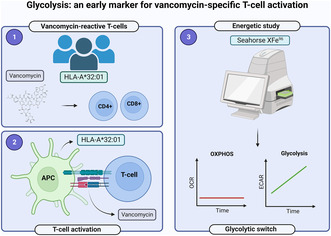

**Key messages**

- CD4+ and CD8+ vancomycin‐specific T‐cell clones undergo glycolytic switching when activated with vancomycin.
- Glycolytic switching of vancomycin‐specific T‐cell clones was dose‐dependent, drug‐specific and HLA restricted.
- T‐cell clones displayed energetic cross‐reactivity with teicoplanin, despite the absence of proliferative and effector cytokine responses.

## 1 INTRODUCTION

Adaptive immune responses are implicated in the pathogenesis of hypersensitivity reactions to a multitude of drug classes including β‐lactam antibiotics, sulfonamides and anticonvulsants.^1^, ^2^, ^3^ T‐cell activation has long been established as a key determinant of cell‐mediated (Type IV) hypersensitivity, with ensuing effector functions resulting in the release of cytotoxic molecules and subsequent tissue damage.^4^, ^5^ However, the metabolic phenotypes that underpin the initiation and propagation of a drug‐specific T‐cell response have not been defined.

Vancomycin is a first‐generation glycopeptide antibiotic used in the treatment of severe infections caused by Gram‐positive bacteria (e.g. methicillin‐resistant *Staphylococcus aureus* (MRSA) and *Clostridium difficile*).^6^ Recently, vancomycin administration has been linked as a causative factor within drug reaction with eosinophilia and systemic symptoms (DRESS), for which pathogenesis is entwined with delayed onset, aberrant T‐cell function.^7^, ^8^ Furthermore, a pharmacogenetic study into the link between vancomycin exposure and DRESS reactions has now shed light on a specific human leukocyte antigen (HLA) association (HLA‐A*32:01), indicating involvement of the adaptive immune system and potential role for the activation of T cells.^9^ Recent work using HLA‐A*32:01 positive healthy human donors has shown the importance of an interaction between vancomycin and HLA‐A*32:01 for T‐cell activation, with T‐cell effector functions evoked by direct, non‐covalent pharmacological interactions between the drug and MHC.^10^

Researchers have recently started to characterise the energetic pathways that underpin immune‐cell activation, such as glycolysis and oxidative phosphorylation (OXPHOS), in an attempt to define the essential metabolic phenotypes necessary for rapid cellular growth and proliferative function. Furthermore, the activation of T cells from inherent resting and naïve states requires metabolic reprogramming in which fuel sources are readily available to cope with energetic demands of clonal expansion. Antigen presentation evokes T‐cell activation via interactions between antigen, antigen presenting cells (APCs) displaying major histocompatibility complexes (MHC) and the T‐cell receptor (TCR). Studies have now demonstrated that ensuing CD4+ and CD8+ T‐cell effector functions are dependent on glycolysis.^11^, ^12^ This form of metabolic reprogramming has been described as an immediate‐early ‘glycolytic switch’ when applied to both de novo and memory T‐cell responses.^13^ To this end, the published literature has broadly focused on characterising metabolic phenotypes within polyclonal T‐cell populations, in addition to energetic changes following artificial (αCD3/28) and peptide‐induced stimulation.^11^ Given the unique pathways involved in the activation of drug‐specific T cells, there is a need to elucidate the metabolic traits and fuel dependence that governs T‐cell responses to drugs within single cell populations. This may provide mechanistic insight into the conditions necessary for recall responses within the context of drug hypersensitivity reactions.

The initial focus of this work was to develop an assay capable of detecting both non‐specific and drug‐specific markers of T‐cell activation, through measurement of the glycolytic fuel pathway using the Seahorse XFe96 Respirometer. These assays enable the measurement of extracellular acidification rates (ECAR) that can be converted to glycolytic ATP production rates (JATPglyc) indicative of glycolytic function. Bioenergetics assays allowed for the study of metabolic signatures associated with the drug‐specific activation process of vancomycin‐reactive CD4+ and CD8+ T‐cell clones (TCCs) generated from healthy donors and hypersensitive patients expressing HLA‐A*32:01, and determined whether metabolic signalling in T cells is exquisitely associated with T‐cell proliferative responses and cytokine release.

## 2 METHODS

### 2.1 PBMC from human subjects

Peripheral blood mononuclear cells (PBMCs) were obtained from healthy, HLA‐genotyped donors naïve for vancomycin exposure, accessed through a biobank of 1200 HLA typed donors.^14^, ^15^ PBMC isolated from three patients with confirmed vancomycin hypersensitivity were acquired following approval the from Local Ethics Committee and with written, informed consent. Samples were also collected after material transfer agreement (MTA) approval. HLA typing (HLA‐A, HLA‐B, HLA‐C, HLA‐DRB1, HLA‐DRB345, HLA‐DQA1, HLA‐DQB1, HLA‐DPA1 and HLA‐DPB1) at 3× resolution was performed by the Histogenetics laboratory (Histogenetics, Ossining, NY).

### 2.2 T‐cell clone generation

PBMCs isolated from healthy donors and vancomycin‐hypersensitive patients were used to generate CD4+ and CD8+ expressing vancomycin‐specific TCCs using a serial dilution protocol.^16^ Drug‐reactive TCCs were subjected to a battery of phenotypic and functional assays to determine pathways of T‐cell activation, processing capacity and cross‐reactive potential, detailed in the Appendix S1.

### 2.3 Glycolytic dependence

Assays were developed and optimised to explore the energetic requirements associated with the activation of single T‐cell populations. Monoclonal drug‐specific TCCs generated from healthy donors and vancomycin‐hypersensitive patients were assessed for glycolytic dependence upon acute activation with model stimulants (phytohaemagglutinin and anti‐CD3 activating antibodies) and vancomycin using the Seahorse XFe96 Respirometer. The oxygen consumption rate (OCR) and ECAR were measured to quantify mitochondrial respiration and glycolysis respectively. Additionally, JATPglyc and JATPox were quantified to allow for the direct assessment of energy production through each metabolic pathway. Energetic function of TCCs with predetermined compound specificity were analysed in quadruplicate, with 3 × 10^5^ TCCs/well required for viable metabolic readouts. Detailed methods information can be found in the Appendix S1.

### 2.4 Statistical analysis

Statistical analysis was performed using GraphPad Prism 9.0 software and data are presented as mean ± SEM. To evaluate T‐cell responses for which data are normally distributed, a student's *t*‐test or one‐way ANOVA was used. For data sets not normally distributed, such as functional T‐cell assays, statistical analysis was performed using a Mann–Whitney *U* test. For both tests, *p < .05 was considered to be the threshold for significance.

## 3 RESULTS

### 3.1 Generation of CD4+ and CD8+ vancomycin‐specific TCCs from HLA‐A*32:01‐positive healthy donors and vancomycin‐hypersensitive patients

**FIGURE 1:**
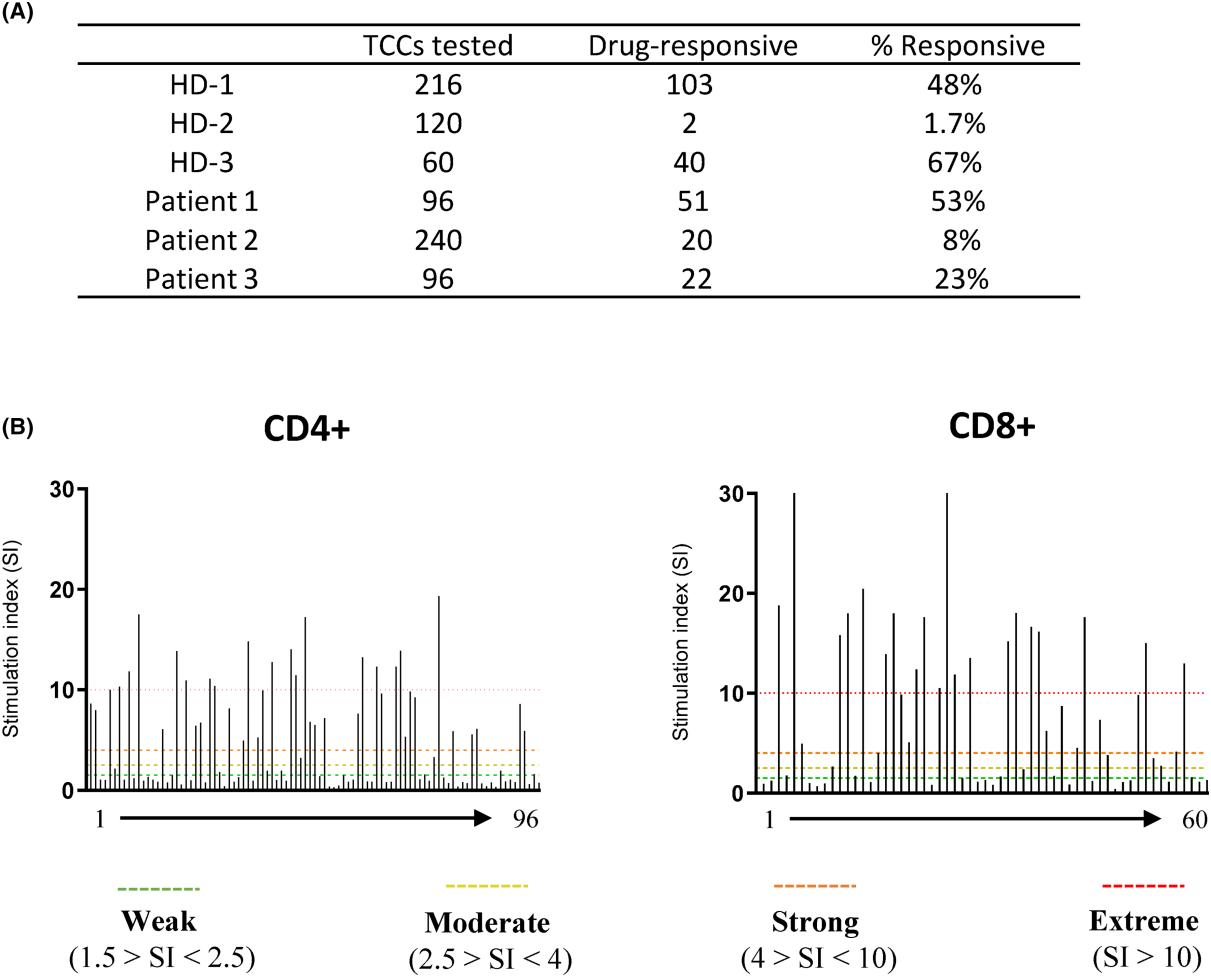
Generation of vancomycin‐specific TCCs from PBMC enriched for CD4+ and CD8+ T cells in healthy donors and vancomycin‐hypersensitive patients expressing HLA‐A*32:01. (A) Number of vancomycin‐responsive TCCs (SI >1.5) after drug rechallenge. TCCs were assessed for drug specificity using proliferation readout [^3^H]. Total numbers of cultures tested for vancomycin specificity shown across healthy donor and patient groups positive for HLA‐A*32:01. (B) Bulk PBMC cultures were seeded at 1 × 10^6^ PBMC/well with graded concentrations of vancomycin (0.1–0.5 mM) for 14 days. T cells were enriched for CD4+ or CD8+‐expressing populations by MACS separation and transferred to 96‐well U‐bottomed plates (1 T cell/well average) with irradiated allogenic PBMC (5 × 10^4^ cells/well) and medium supplemented with IL‐2 (200 U/mL) and PHA (5 μg/mL). Test cultures were rechallenged with vancomycin (0.5 mM) or medium only, in the presence of irradiated autologous EBV‐transformed B cells (1 × 10^4^ cells/well). Testing was performed over four wells in duplicate conditions and cultures were incubated for 48 h and pulsed with tritiated [^3^H]‐thymidine (0.5 μCi/well) for 16 h before scintillation counting. Readouts were given as counts per min (cpm) and SI values for each test culture were interpreted as either weak (1.5 > SI <2.5), moderate (2.5 > SI <4), strong (4 > SI < 10) or extreme (SI > 10). Data are shown for representative CD4+ and CD8+‐enriched populations.

Vancomycin‐responsive CD4+ and CD8+ expressing TCCs were generated from three healthy donors positive for HLA‐A*32:01 and three hypersensitive patients positive for HLA‐A*32:01 (Figure 1). As an example, a total of 103 TCC were stimulated to proliferation with vancomycin from 216 test cultures, with 35 categorised as ‘extreme’ responders giving a drug‐specific stimulation index of >10‐fold above the media control (Figure 1B). Multiple drug‐specific TCCs were generated from all three vancomycin‐hypersensitive patients. For one patient (patient 1) >50% of TCCs exhibited specificity for vancomycin after drug rechallenge (Figure 1A). A further 20% of these TCCs were categorised as ‘extreme’ responders.

A total of 24 vancomycin‐specific TCCs expressing both CD4+ and CD8+ phenotypes were expanded via repetitive mitogen‐driven stimulation. Eight TCCs were deemed suitable for energetic profiling after cell numbers passed a predetermined threshold value of approximately 1 × 10^7^ cells per TCC. CD8+ vancomycin‐reactive TCCs were used to assess energetic responses to model T‐cell stimulants and vancomycin at graded dosage. CD4+ TCCs were used to study the HLA restricted nature of energetic switching. Additionally, energetic differences between CD4+ and CD8+ drug‐specific T cells were evaluated using single cell populations (Figure S2).

### 3.2 Model stimulants facilitate a transition towards glycolytic fuel pathways in CD8+ vancomycin‐specific TCCs

**FIGURE 2:**
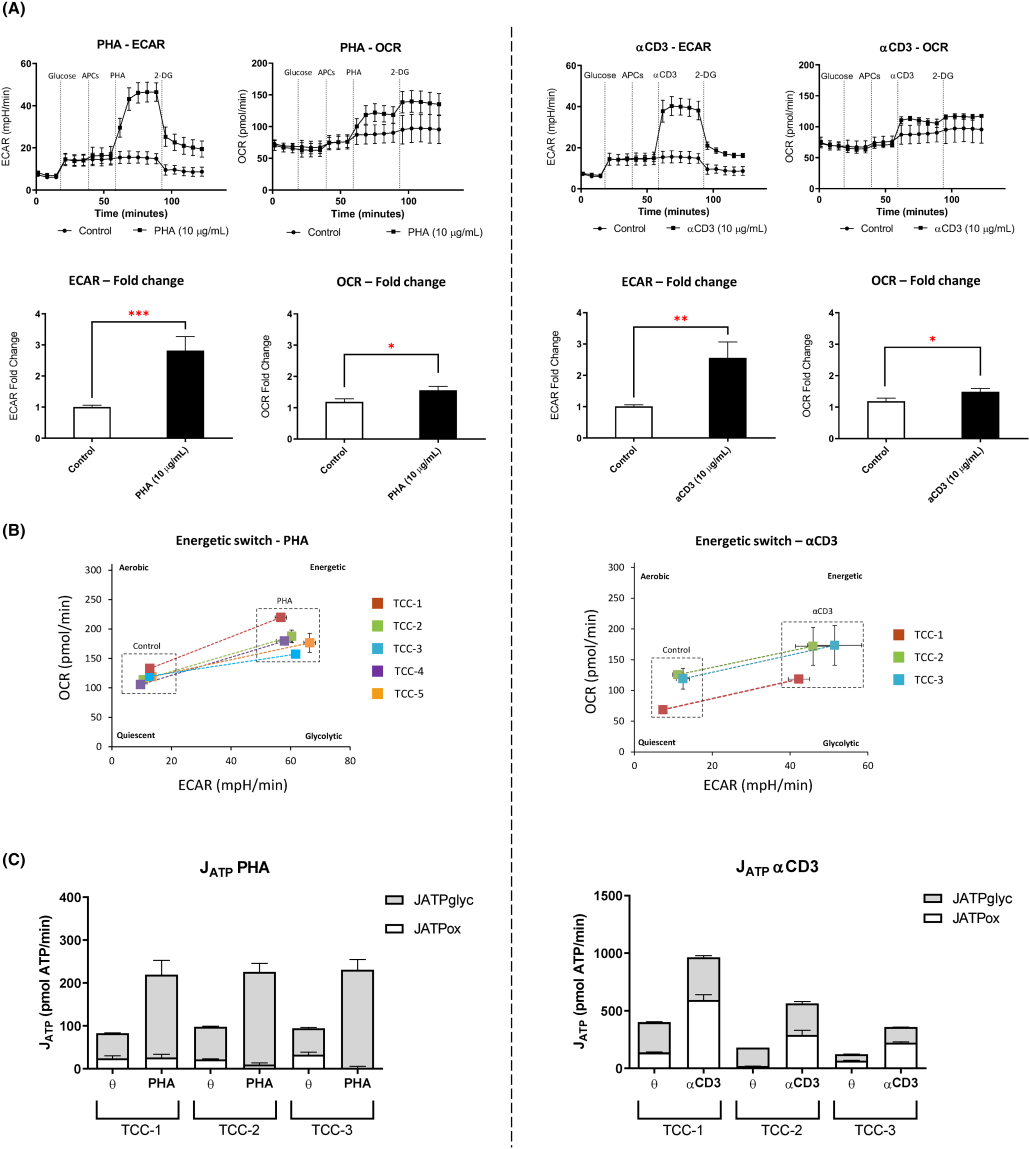
Glycolysis stress test of model T‐cell stimulants in drug‐specific TCCs. (A) Vancomycin‐specific TCCs (3 × 10^5^) were sequentially exposed to D‐glucose (25 mM), autologous APCs (5 × 10^4^) and either PHA (10 μg/mL) or αCD3 (10 μg/mL), followed by a final injection of 2‐DG (25 mM). Data are shown for a representative TCC and given by ECAR (mpH/min) and OCR (pmol/min) readouts. Statistical significance was determined using a student's *t*‐test (*p < .05, **p < .01 and ***p < .0001). (B) Energetic shifting (OCR vs. ECAR) following acute injection of model stimulants. Data are shown for five representative TCCs (PHA injection) and three TCCs (αCD3 injection). (C) JATPglyc and JATPox values (pmol ATP/min) of TCCs (*n* = 3) after acute injection of PHA or αCD3.

Glucose injection increased ECAR values, representing basal levels of TCC glycolytic function. Exposure to autologous‐irradiated antigen presenting cells (APCs) did not result in any detectable energetic changes. Assays assessing the utility of PHA for ECAR‐dependent T‐cell activation revealed TCCs exhibited elevated ECAR levels within 10 min of exposure. PHA induced statistically significant increases in both ECAR and oxygen consumption rate (OCR) levels, but the impact on ECAR was substantially higher with a 63% elevation detected compared to basal levels of glycolytic function (Figure 2A). Metabolic phenotyping of all TCCs revealed a distinct shift from a quiescent state towards a more energetic and glycolytic state upon mitogen‐driven T‐cell activation (Figure 2B).

Similar results were observed after stimulation of drug‐specific TCCs with αCD3. ECAR dependence was found to be favoured over OCR following acute stimulation with αCD3 antibodies (Figure 2A). Analysis of the metabolic phenotypes of these cellular populations via the comparison of OCR and ECAR values before and after stimulation with αCD3 revealed a similar shift from quiescent and inactivated states towards a more energetic and glycolytic state upon TCR‐mediated activation (Figure 2B). When profiling glycolytic dependence of drug‐specific TCCs after αCD3 exposure, the addition of αCD28 antibodies did not further influence the metabolic readout (Figure S1).

To calculate the rate of ATP production through glycolysis and OXPHOS after exposure to model T‐cell stimulants, ECAR and OCR values were converted to JATPglyc and JATPox as per the method of Mookerjee et al.^17^ Calculation of bioenergetic rates of ATP production revealed that PHA induced a strong energetic shift away from OXPHOS and towards glycolysis (JATPglyc). After stimulation with αCD3, the rate of ATP production through OXPHOS (JATPox) was observed to increase alongside JATPglyc (Figure 2C).

### 3.3 Activation of CD8+ vancomycin‐reactive TCCs shows dependence for glycolysis in a dose‐dependent and drug‐specific manner

**FIGURE 3:**
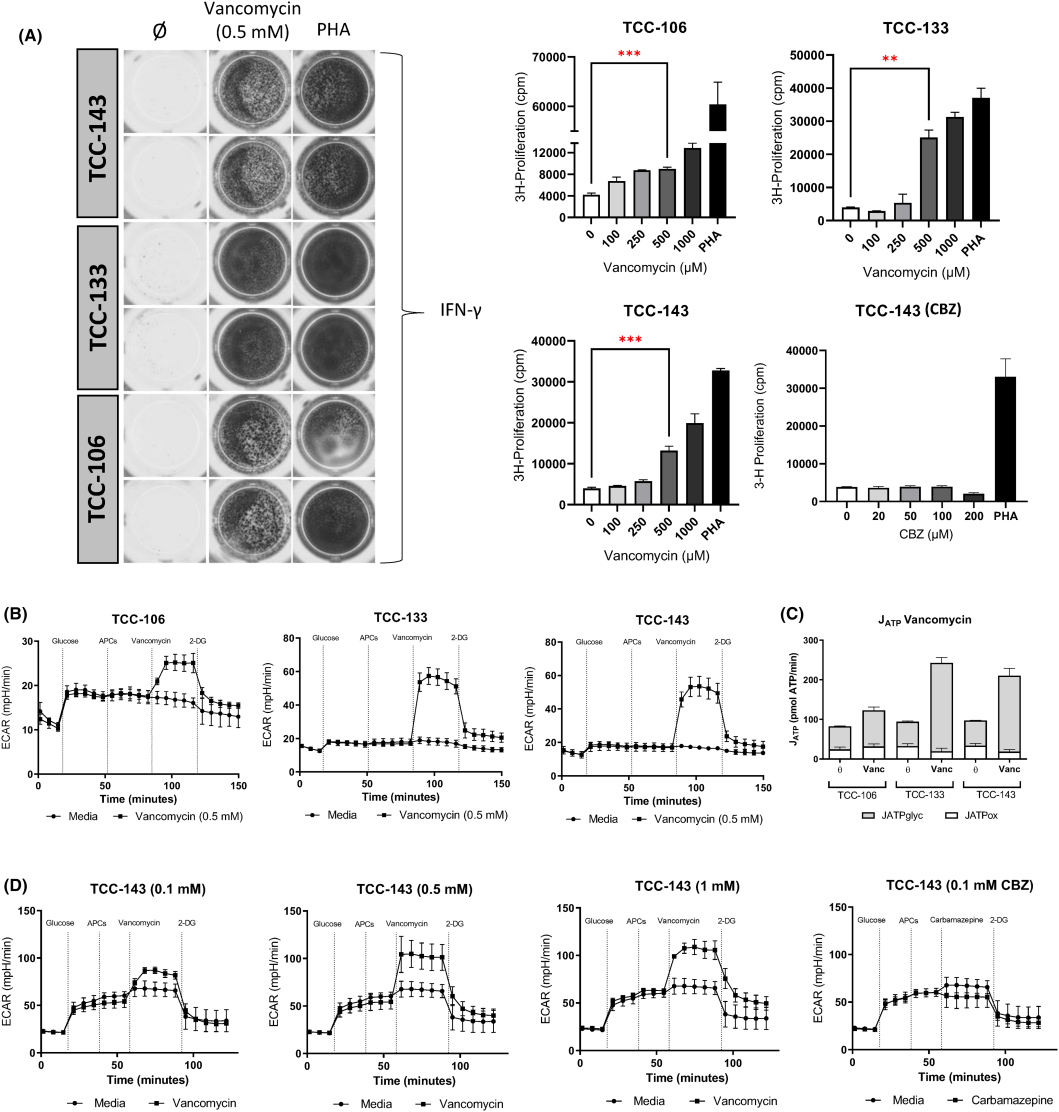
Glycolytic activation of CD8+ vancomycin‐specific TCCs and conventional T‐cell readouts. (A) Cytokine release from CD8+ vancomycin‐specific TCCs. TCCs (5 × 10^4^) were co‐cultured with autologous EBV‐transformed B cells (1 × 10^4^ cells) and either vancomycin (0.5 mM), medium or PHA (10 μg/mL). Cytokine secretion (IFN‐γ) was measured after 48 h incubation by ELISpot. To assess the drug‐specific nature of proliferative responses, vancomycin‐specific TCCs (5 × 10^4^) were co‐cultured with autologous EBV‐transformed B cells (1 × 10^4^ cells) and either vancomycin (0–1 mM) or carbamazepine (0–200 μM). Cells were incubated for 48 h before pulsation with tritiated [^3^H]‐thymidine (0.5 μCi/well) for 16 h. Proliferation was determined by [^3^H]‐incorporation and interpreted as cpm values. Statistical significance was determined using a student's *t*‐test (**p < .01, ***p < .001). (B) Glycolysis stress test incorporating vancomycin exposure (TCCs; *n* = 3). Vancomycin‐specific TCCs were sequentially exposed to D‐glucose (25 mM), APCs (5 × 10^4^) and vancomycin (0.5 mM) followed by a final injection of 2‐DG (25 mM). (C) JATPglyc and JATPox values (pmol ATP/min) of vancomycin‐specific TCCs (*n* = 3) after acute vancomycin exposure. (D) Glycolysis stress test at graded vancomycin concentrations (0.1, 0.5 and 1 mM) and carbamazepine (100 μM).

Three representative CD8+ expressing TCCs were assessed for cytokine secretion, proliferation and glycolytic switching after vancomycin treatment. Each TCC was found to release IFN‐γ and proliferate in a dose‐dependent manner after drug exposure (Figure 3A). Drug‐specific T‐cell activation was observed to be a highly glycolytic process (Figure 3B). All three TCCs (TCC‐106, TCC‐133 and TCC‐143) exhibited glycolytic switching immediately after exposure to autologous irradiated APCs and optimal concentrations of vancomycin (0.5 mM). Treatment with 2‐DG successfully reversed the ECAR dependence observed, indicating that glycolysis was the primary metabolic pathway utilised by drug‐activated T cells (Figure 3B). Analysis of ATP production rates through glycolysis (JATPglyc) further confirmed the specificity of the energetic switch in favour of glycolysis (Figure 3C). Energetic comparison between CD4+ and CD8+ TCCs suggests an increased glycolytic capacity in CD4+ expressing single cell populations following vancomycin rechallenge (Figure S2).

Due to high levels of clonal expansion, one CD8+ vancomycin‐reactive TCC (TCC‐143) was further assessed for the presence of both dose‐dependent and drug‐specific energetic responses to vancomycin and the structurally unrelated compound carbamazepine (CBZ; Figure 3D). Acute vancomycin exposure resulted in a dose‐dependent increase between 0.1 mM and 0.5 mM vancomycin concentrations within the ECAR readout (Figure 3D). No detectable change within ECAR readout was observed when the TCC was exposed to CBZ at the optimal T‐cell stimulatory dose. These data aligned with proliferative responses of vancomycin‐specific TCCs when tested for cross‐reactivity with CBZ at graded drug concentrations (Figure 3A).

**FIGURE 4:**
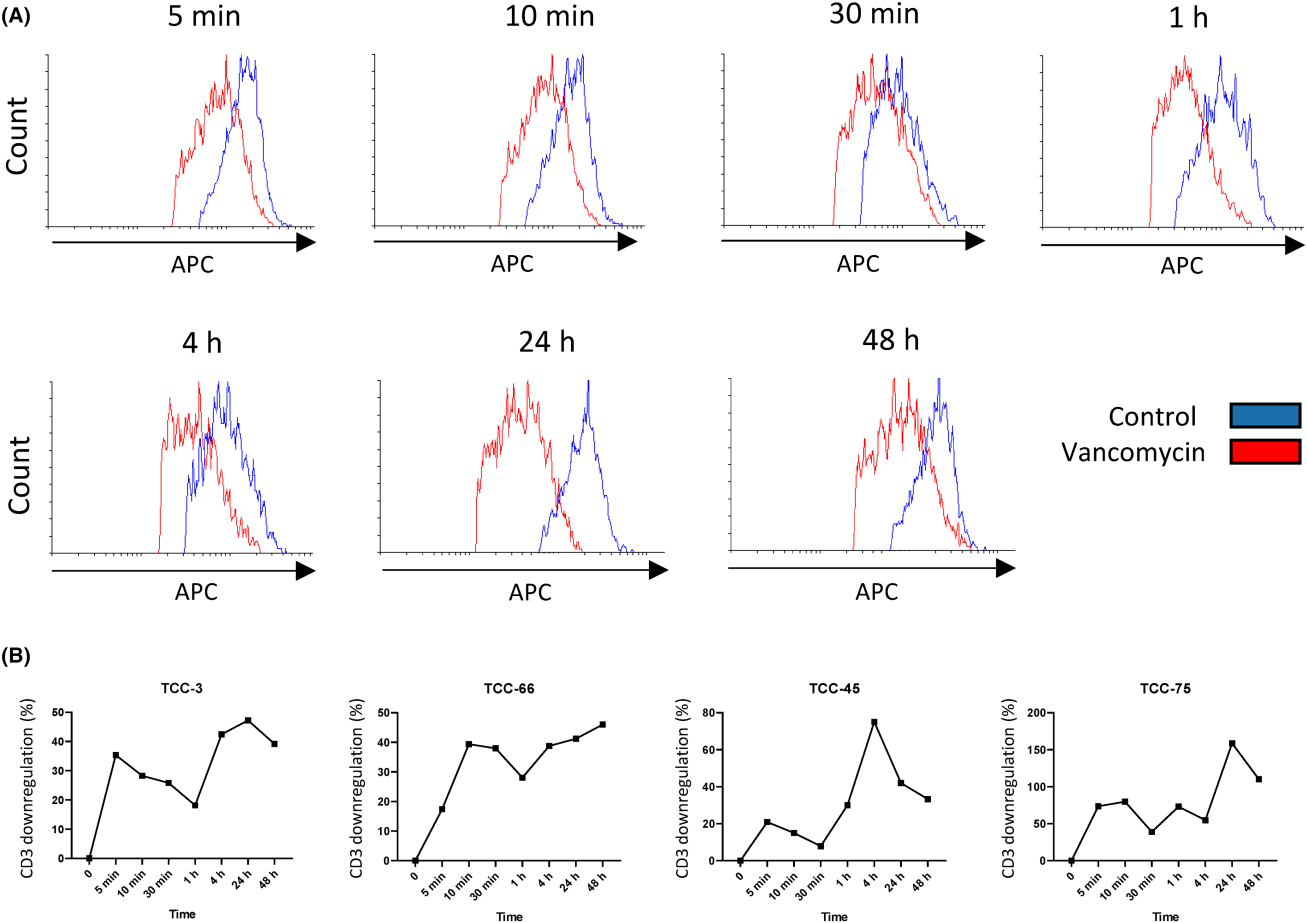
CD3 downregulation of CD4+ drug‐specific TCCs after vancomycin treatment. (A) T cells (5 × 10^4^) with predetermined drug specificity were pulsed with vancomycin (0.5 mM) at multiple time points (5 min, 10 min, 30 min, 1 h, 4 h, 24 h and 48 h) or cell culture medium only. Activated cultures were stained with anti‐CD3 (APC) antibodies and samples were analysed for 10^4^ using a FACS‐Canto II instrument. (B) CD3 downregulation after vancomycin exposure (TCCs; *n* = 4). CD3 downregulation was interpreted as a percentage between control and drug‐exposed cultures. Unstained and untreated cultures were set up in parallel for comparison and samples were analysed using a FACS‐Canto II instrument integrated with FACS DIVA operating software and phenotypic analysis was carried out using Flowing 2 software to calculate CD3 downregulation (%) of each TCC after pulsation with either vancomycin or medium for multiple time points.

CD4+ vancomycin‐reactive TCCs were assessed for CD3 downregulation (a sensitive marker of TCR triggering) after stimulation with vancomycin at time points correlating with glycolytic activation. CD3 expression was found to be downregulated 5 min after vancomycin exposure, and at each subsequent time point studied (Figure 4).

### 3.4 CD4+ TCCs with vancomycin specificity display APC‐dependent HLA restricted activation

**FIGURE 5:**
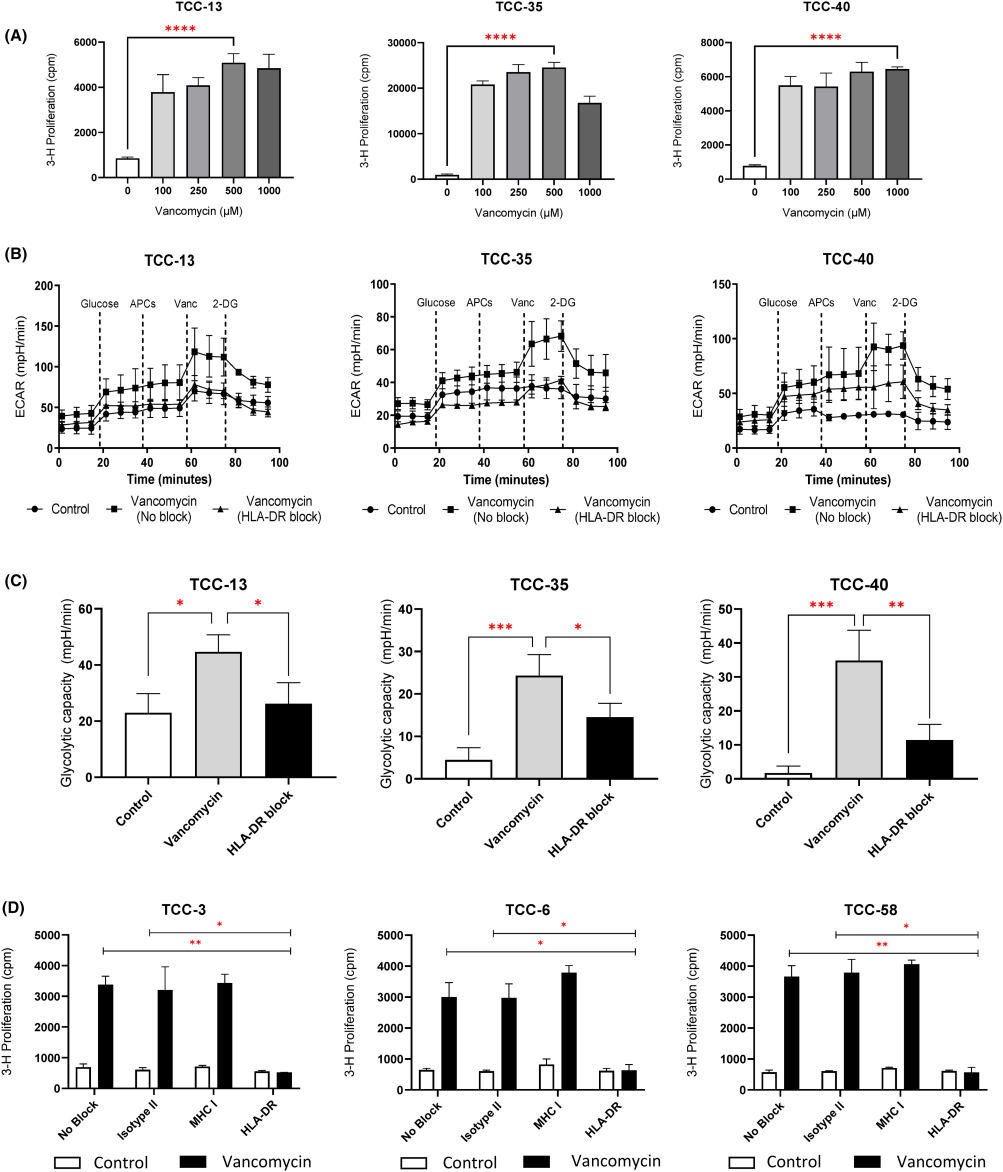
Glycolysis stress test in CD4+ vancomycin‐specific TCCs assessing energetic response to vancomycin after HLA‐DR blockade. (A) Proliferative responses of CD4+ vancomycin‐specific TCCs. TCCs (5 × 10^4^) were co‐cultured with autologous EBV‐transformed B‐cells (1 × 10^4^ cells) and vancomycin (0–1 mM). Cells were incubated for 48 h before pulsation with tritiated [^3^H]‐thymidine (0.5 μCi/well) for 16 h. Proliferation was determined by [^3^H]‐incorporation and interpreted as cpm values. Statistical significance was determined using a student's *t*‐test (****p < .0001). (B) TCCs (3 × 10^5^) were blocked with HLA‐DR antibodies (10 μg/mL) for 1 h. TCCs were sequentially treated with D‐glucose (25 mM), APCs (5 × 10^4^) and either vancomycin (0.5 mM) or medium followed by a final injection of 2‐DG (25 mM). (C) Glycolytic capacity (mpH/min) of CD4+ vancomycin‐specific TCCs after acute drug exposure and HLA‐DR blockade. Statistical significance was established using one‐way ANOVA (*p < .01, **p < .001 and ***p < .001). (D) Proliferative responses to vancomycin after HLA‐DR blockade. TCCs (5 × 10^4^) were co‐cultured with irradiated autologous EBVs (1 × 10^4^) and either HLA Class I (10 μg/mL), HLA‐DR (10 μg/mL) or corresponding IgG2 isotype controls (10 μg/mL) for 1 h. Blocked cultures were treated with vancomycin (0.5 mM) or medium for 48 h before pulsation with tritiated [^3^H]‐thymidine and scintillation counting. Statistical significance was determined using a Mann–Whitney *U* test (*p < .05, **p < .01).

Vancomycin‐responsive CD4+ TCCs proliferate in a dose‐dependent manner after drug rechallenge (Figure 5A) and displayed similar rapid glycolytic responses following acute vancomycin exposure (Figure 5B). Additionally, the glycolytic capacity of these cells was strongly enhanced after vancomycin treatment (Figure 5C). These TCCs were subsequently used to explore the importance of antigen presenting cells and HLA dependency of the energetic readout. The CD4+ TCCs displayed abrogated ECAR responses after HLA‐DR blockade and vancomycin exposure, when compared to TCCs assessed in the absence of HLA‐DR blockade (Figure 5B). These data, indicating that vancomycin presentation via HLA‐DR is necessary for the induction of a glycolytic response, are concordant with HLA‐DR‐restricted proliferative responses to vancomycin in a panel of TCCs generated from the same patient (Figure 5D). HLA class I‐restricted activation of vancomycin‐specific CD8+ TCCs (*n* = 3) was demonstrated in T‐cell proliferation assays prior to metabolic assessment (isotype control cpm, 2.9 × 10^4^; MHC class I block cpm, 3.3 × 10^3^; MHC class II block cpm, 2.5 × 10^4^). To assess APC functionality, CD8+ TCCs were exposed to both 50 and 100 μM vancomycin in the presence and absence of antigen presenting cells. Glycolytic switching of TCCs was only observed in the presence of autologous APCs (Figure S3).

### 3.5 CD8+ TCCs display immediate ECAR escalation, but are not stimulated to proliferate or secrete IFN‐γ, with the glycopeptide teicoplanin

**FIGURE 6:**
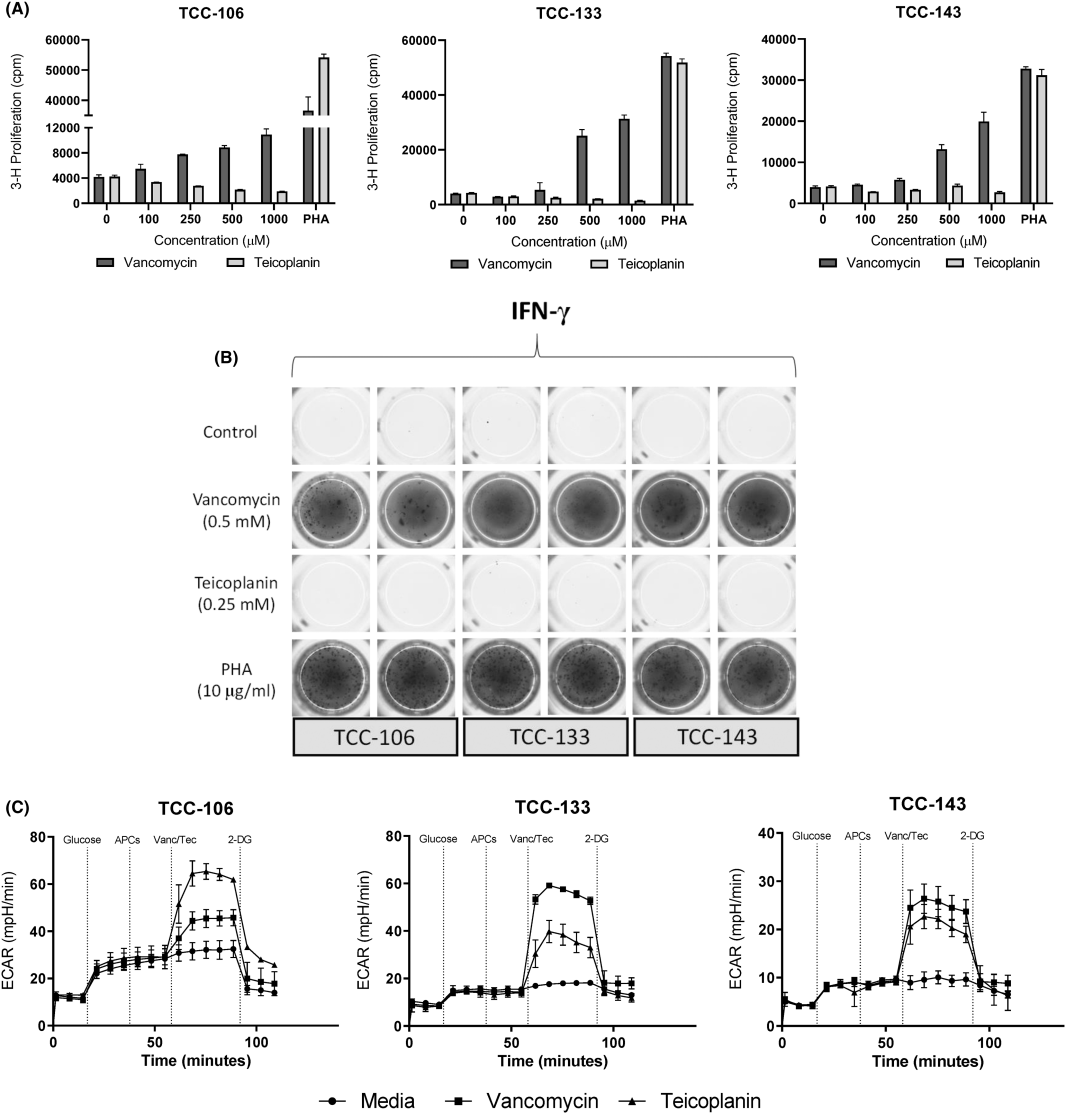
Energetic cross‐reactivity between CD8+ vancomycin‐specific TCCs and teicoplanin. (A) Proliferative cross‐reactivity study of vancomycin‐specific TCCs generated from healthy donor PBMC positive for HLA‐A*32:01 expression (*n* = 3). TCCs (5 × 10^4^) were co‐cultured with irradiated autologous EBV‐transformed B cells (1 × 10^4^ cells) and either vancomycin (0–1000 μM), teicoplanin (0–1000 μM) or PHA (10 μg/mL). Cultures were incubated for 48 h (37°C, 5% CO_2_) prior to pulsation with tritiated [^3^H]‐thymidine and scintillation counting (B) Assessment of glycopeptide cross‐reactivity by cytokine release. TCCs (5 × 10^4^) were co‐cultured with autologous EBV‐transformed B cells (1 × 10^4^ cells) and either vancomycin (0.5 mM), teicoplanin (0.25 mM), medium or PHA (10 μg/mL). IFN‐γ release was detected after 48 h incubation by ELISpot. (C) Vancomycin‐specific TCCs were assessed for cross‐reactivity with teicoplanin by glycolysis stress test assay after acute injection of vancomycin or teicoplanin. TCCs were sequentially treated with D‐glucose (25 mM), APCs (5 × 10^4^) and either vancomycin (0.5 mM), teicoplanin (0.25 mM) or Seahorse XF base medium followed by a final injection of 2‐DG (25 mM).

CD8+ expressing TCCs displayed proliferative responses and IFN‐γ secretion with vancomycin, but not teicoplanin (Figure 6A,B). Interestingly, cross‐reactivity was detected when profiling the energetic parameters of T‐cell activation after acute exposure of the same TCCs to vancomycin and teicoplanin (Figure 6C). Immediate ECAR escalation was observed following exposure of vancomycin and teicoplanin in 3/3 TCCs profiled for cross‐reactivity, with 2‐DG inducing energetic blockade.

## 4 DISCUSSION

Vancomycin‐induced DRESS can be severe and limit the choice of antibiotics for Gram‐positive bacterial infections.^18^, ^19^ The recent discovery of (i) HLA‐A*32:01 carriage as a susceptibility factor for vancomycin‐induced DRESS^9^ and (ii) the detection of vancomycin‐responsive T cells in patients with DRESS and healthy HLA‐A*32:01+ donors^10^ highlights the importance of adaptive immunity in disease pathogenesis. In this study, vancomycin‐reactive CD4+ and CD8+ TCCs were successfully generated from healthy donors and patients with vancomycin‐induced DRESS (Figure 1). Vancomycin specificity was confirmed using proliferation and IFN‐γ secretion as readouts, prior to energetic profiling and assessment of cross‐reactivity to determine whether energetic signalling is linked to other T‐cell activation events.

Empirical evidence now exists detailing metabolic switching to both glycolysis and Warburg metabolism following the activation of resting, memory and naïve T cells, with intrinsic differences now apparent between the metabolic activities of CD4+ and CD8+ expressing T cells following TCR stimulation.^11^, ^20^ As circulating lymphocytes exit quiescence following antigen exposure, there is evidence that glycolytic precursors within glucose metabolism are upregulated to support rapid growth and clonal expansion.^21^, ^22^ Applications utilising metabolic signatures of cloned T cells, such as glycolytic switching after stimulation, may permit the comparison of metabolic activity on a single cell level and serve as an early marker for the detection of drug‐specific memory T‐cell response pathways. In this study, we focused on vancomycin‐responsive T cells as (i) vancomycin‐induced DRESS is clinically important and cross‐reactive T‐cell responses to glycopeptide antibiotics such as vancomycin and teicoplanin have recently been described,^10^, ^23^ (ii) the drug stimulates both CD4+ and CD8+ T cells and (iii) the pathway of vancomycin‐specific T‐cell activation is ill‐defined.

In order to delineate the presence of metabolic adaptation that underpins the ability of drug‐specific TCCs to enter a glycolytic‐like state, it was necessary to optimise and develop assays capable of measuring the energetic parameters of T‐cell activation following acute stimulation. Transformed B‐cell lines have been shown to be proficient at antigen presentation culminating in the elicitation of antigen‐specific T‐cell responses.^24^ Consequently, incorporation of B cells (as APCs) within the assay system for the measurement of energetic parameters associated with T‐cell activation represented a key component of the assay development process. APCs were exposed to gamma irradiation, to inhibit proliferation whilst still retaining functionality in terms of antigen presentation and acutely injected within the assay at similar ratios described in the conduction of conventional functional T‐cell assays.^2^ Importantly, the addition of APCs to the assay systems induced no effect on basal energetic readouts (Figures 2 and 3). For the detection of drug‐specific T‐cell responses manifesting as a visible ‘switch’ between metabolic pathways, positive controls in the form of model T‐cell activators with well‐defined stimulatory properties were introduced and optimised for use within glycolysis stress test assays (Figure 2). Firstly, PHA isolated from *Phaseolus vulgaris* was shown to induce a strong fluxing towards glycolytic dependence in preference to OXPHOS, as expected of the transition between T cells in quiescent and activated states. Indeed, PHA activates human T cells in a non‐specific manner to induce proliferation and is widely deployed within functional T‐cell assays for validation purposes.^25^

To develop the assay for the assessment of drug‐specific T‐cell activation, it was important to assess energetic function after stimulation with compounds possessing greater physiological relevance. As a consequence, both CD3 and CD28 activating antibodies were studied in isolation and co‐operatively to elucidate the impact of TCR‐mediated stimulation on the activation profile of drug‐specific TCCs. CD3 and CD28 are typically amalgamated within functional assays attempting to induce polyclonal T‐cell activation and cellular expansion.^26^ In vitro applications attempt to mimic both signal 1 and signal 2 interactions typically provided through interplay between the APC and TCR for which an activation threshold is determined.^27^ Integration of both CD3 and CD28 activating antibodies revealed no discernible energetic advantage when compared to the activity of anti‐CD3 in isolation (Figure S1). From this, it can be deduced that fuel pathway dependence, for which glycolysis was implicated, was influenced by the presence of signal 1 but not signal 2. In this way, glycolytic switching measured within the assay system was sensitive to interactions between antigen and TCR, mimicked by the addition of anti‐CD3, but not to any co‐stimulation provided by anti‐CD28 for which T‐cell survival would be promoted and a proliferative effect induced.^28^ This may be due to TCCs possessing a memory phenotype, therefore negating the requirement for additional, costimulatory signalling needed for T‐cell activation.

To complement the body of work detailing fuel dependency after artificial stimulation for which a glycolytic shift has been implicated, we aimed to determine if ECAR measurement and JATPglyc could be used as a metabolic signature for drug‐specific T‐cell activation.^13^, ^29^ Vancomycin‐reactive TCCs expressing CD8+ phenotypes with predetermined drug specificity were found to exhibit metabolic switching accentuated by an increased dependence on glycolysis after acute vancomycin exposure (Figure 3B). This was evidenced by an increase within JATPglyc rates after drug stimulation, directly implicating glycolysis as the primary metabolic pathway involved (Figure 3C). Furthermore, the energetic changes detected with stress‐based assays were found to be dose‐dependent and drug‐specific, concordant with assays performed in parallel detailing proliferative responses and cytokine release (Figure 3A,D). This was important for validation of the assay and provided affirmation that the observed glycolytic response was vancomycin‐specific and not caused by cellular stress, as indicated by the absence of any detectable response following the addition of a structurally unrelated compound and the addition of vancomycin in the absence of APCs. Analysis of maximal ECAR values and comparative study between T‐cell subpopulations suggests CD4+‐expressing TCCs possess a greater capacity for glycolytic induction than CD8+ TCCs (Figure S2). These data align with similar studies assessing both glycolytic dependence and the expression of glycolytic enzymes such as Hexokinase II, for which CD4+ T cells have been observed to possess a superior glycolytic phenotype.^11^

It was necessary to confirm the presence of functional antigen presentation within the glycolysis stress test to verify both physiological relevance and provide a degree of synchronisation between metabolic readouts and in vitro T‐cell assays. We have demonstrated that APCs are required for CD8+ vancomycin‐specific T‐cell activation. Additionally, the blockade of HLA‐DR in experiments with vancomycin‐specific CD4+ TCCs resulted in abrogation of glycolytic dependence after vancomycin exposure (Figure 5A). Therefore, antigen presentation capable of eliciting energetic responses to vancomycin has been successfully modelled with HLA interactions clearly visible. In this study, large numbers of CD4+ vancomycin‐specific TCCs were generated from hypersensitive patients, despite the documented HLA class I association. There are number of potential reasons for this phenomenon. Firstly, it must be stated that numerous studies have indicated that DRESS pathogenesis can mediated by both CD4+ and CD8+ T‐cell responses and although a genetic association exists between vancomycin and HLA‐A*32:01, only 20% of individuals expressing the risk allele will develop DRESS after vancomycin treatment.^9^, ^30^ This data aligns with T‐cell cloning experiments performed with both dapsone and flucloxacillin‐hypersensitive patients. Dapsone (HLA‐B*13:01) and flucloxacillin (HLA‐B*57:01) have been strongly associated with expression of HLA class I molecules; however, studies have shown drug‐specific CD4+ TCCs can be generated in abundance.^31^, ^32^ Indeed, the pathogenesis of drug hypersensitivity reactions often involves collective input from both CD4+ and CD8+ T cells, with the notable exception of abacavir for which a CD8+ T‐cell response is mounted in isolation.^33^ Previous work has successfully demonstrated HLA class I‐restricted activation of CD8+ vancomycin‐specific TCCs within proliferation‐based assays.^10^ However, further studies are on‐going to explore the energetics of HLA class I restriction of CD8+ TCCs and indeed the importance of HLA‐A*32:01 in drug binding and T‐cell activation.

Finally, it was important to corroborate the immediacy of the observed metabolic traits that signify T‐cell activation with surface marker expression at time points relevant to the glycolysis stress test assay. Surface CD3 was found to be rapidly (within minutes) internalised on TCCs after stimulation with vancomycin at time points correlating with glycolytic activation (Figure 4). Downregulation of CD3 surface receptor expression can be used as a reliable maker of T‐cell activation following drug: TCR ligation.^34^, ^35^ These data suggest that vancomycin interacts directly with surface MHC and the TCR of TCCs to trigger effector functions. This agrees with our previous work showing that antigen processing is not required for the activation of T cells with vancomycin.^10^ Vancomycin has a large structure with a molecular weight (MW = 1449.3 g/mol). Thus, it is important that future structural studies investigate the nature of the vancomycin interaction with MHC and MHC‐binding peptides.

Evidence firmly points towards elevated glucose uptake and the induction of an early immediate glycolytic switch, necessary to keep pace with the energetic demand of T‐cell activation.^13^, ^36^, ^37^ More recently, the role of glycolytic metabolism within T‐cell activation has been further affirmed for a variety of cellular responses, most notably when studying graft‐versus‐host disease for which elevated and sustained levels of glycolysis have been associated with allogenic T‐cell responses.^38^, ^39^ With respect to fuel pathway dependence following immune stimulation, it follows that the cellular glycolytic potential of drug‐reactive T cells may govern the degree of proliferative and functional response, if not ultimately the nature of such responses. Indeed, it is possible that this phenomenon may underpin cellular fate, either towards elicitation and hypersensitive progression or in favour of tolerance if fuel requirements for full activation are unmet. Importantly, our data demonstrate that an early glycolytic switch is both APC and MHC‐dependent, with binding interaction sufficient for metabolic output and the requirement for co‐stimulatory signalling negated (Figure 5). Furthermore, the adapted glycolysis stress test assay displays utility for the detection of initial drug‐TCR interaction. This is evidenced by the ‘energetic’ cross‐reactivity observed after CD8+ vancomycin‐specific TCCs are exposed to teicoplanin, despite the absence of proliferative cross‐reactivity, (Figure 6). It is possible that the lack of a functional T‐cell response may be impacted by partial, low affinity cross‐reactivity, however, this phenomenon was observed in TCCs with a stronger glycolytic output after teicoplanin exposure (TCC‐106). In this case, we postulate that engagement between teicoplanin, MHC and TCR prepares the T cell for downstream effector function but crucial signalling pathways remain absent for full T‐cell activation to commence.

In summary, access to cloned T cells from healthy human donors and patients with vancomycin hypersensitivity allowed for an in‐depth study of the metabolic signatures associated with mitogen, anti‐CD3/CD28 and drug‐specific activation of CD4+ and CD8+ T cells. Glycolytic activation of TCC with vancomycin was dose‐dependent and HLA restricted. Although metabolic signalling in T cells appears to be a requirement for T‐cell proliferative responses and cytokine release, cross‐reactivity data with teicoplanin indicate that energetic switching is an upstream event and may be seen in the absence of other activation parameters (e.g. proliferation, cytokine release). Future work in this area will explore the correlation between reaction severity and strength of glycolysis in patients presenting with hypersensitivity to multiple drug classes, enabling characterisation of the metabolic processes involved across all pathways of T‐cell activation (hapten hypothesis, pharmacological interaction concept and altered peptide repertoire models). Moving forward, a deeper understanding of the interplay between the molecular and energetic events that determine the fate of stimulated T cells will be crucial to uncovering additional factors that determine the susceptibility to hypersensitivity, for which HLA involvement is now known to be one of the several contributing factors.

## AUTHOR CONTRIBUTIONS

JG conducted the biological experiments. JG, SH and RJ contributed to conceptualisation and experimental design. AG, MSK, EP and MAJ collected the vancomycin patient samples and contributed to the project design. DJN, CB, AC and MP supervised the project. CB (AstraZeneca) was an industry supervisor of JG; no financial support was received in relation to this work. JG analysed the data and drafted the article. All authors reviewed the article.

## FUNDING INFORMATION

JG was a PhD student that received funds from the BBSRC (BB/M011186/1). The project also received funding from an MRC project grant (MR/X00094X/1).

## CONFLICT OF INTEREST STATEMENT

MP has received partnership funding for the following: MRC Clinical Pharmacology Training Scheme (co‐funded by MRC and Roche, UCB, Eli Lilly and Novartis). He has developed an HLA genotyping panel with MC Diagnostics, but does not benefit financially from this. MP and DJN are part of the IMI Consortium ARDAT (www.ardat.org).

## Supporting information


Figure S1

Appendix S1

## Data Availability

Data sets related to this article are available upon request from the corresponding author.

## References

[1] Solensky R . Hypersensitivity reactions to beta‐lactam antibiotics. Clin Rev Allergy Immunol. 2003;24(3):201‐220. 12721392 10.1385/CRIAI:24:3:201

[2] Naisbitt DJ , Britschgi M , Wong G , et al. Hypersensitivity reactions to carbamazepine: characterization of the specificity, phenotype, and cytokine profile of drug‐specific T cell clones. Mol Pharmacol. 2003;63(3):732‐741. 12606784 10.1124/mol.63.3.732

[3] Naisbitt DJ , Gordon SF , Pirmohamed M , et al. Antigenicity and immunogenicity of sulphamethoxazole: demonstration of metabolism‐dependent haptenation and T‐cell proliferation in vivo. Br J Pharmacol. 2001;133(2):295‐305. 11350866 10.1038/sj.bjp.0704074 PMC1572782

[4] Nassif A , Bensussan A , Dorothee G , et al. Drug specific cytotoxic T‐cells in the skin lesions of a patient with toxic epidermal necrolysis. J Invest Dermatol. 2002;118(4):728‐733. 11918724 10.1046/j.1523-1747.2002.01622.x

[5] Yawalkar N , Hari Y , Frutig K , et al. T cells isolated from positive epicutaneous test reactions to amoxicillin and ceftriaxone are drug specific and cytotoxic. J Invest Dermatol. 2000;115(4):647‐652. 10998137 10.1046/j.1523-1747.2000.00105.x

[6] Watanakunakorn C . Treatment of infections due to methicillin‐resistant Staphylococcus aureus. Ann Intern Med. 1982;97(3):376‐378. 7114635 10.7326/0003-4819-97-3-376

[7] Bocquet H , Bagot M , Roujeau JC . Drug‐induced pseudolymphoma and drug hypersensitivity syndrome (drug rash with eosinophilia and systemic symptoms: DRESS). Semin Cutan Med Surg. 1996;15(4):250‐257. 9069593 10.1016/s1085-5629(96)80038-1

[8] Walsh SA , Creamer D . Drug reaction with eosinophilia and systemic symptoms (DRESS): a clinical update and review of current thinking. Clin Exp Dermatol. 2011;36(1):6‐11. 21143513 10.1111/j.1365-2230.2010.03967.x

[9] Konvinse KC , Trubiano JA , Pavlos R , et al. HLA‐A*32:01 is strongly associated with vancomycin‐induced drug reaction with eosinophilia and systemic symptoms. J Allergy Clin Immunol. 2019;144(1):183‐192. 30776417 10.1016/j.jaci.2019.01.045 PMC6612297

[10] Ogese MO , Lister A , Gardner J , et al. Deciphering adverse drug reactions: in vitro priming and characterization of vancomycin‐specific T‐cells from healthy donors expressing HLA‐A*32:01. Toxicol Sci. 2021;183:139‐153. 34175955 10.1093/toxsci/kfab084 PMC8404995

[11] Jones N , Cronin JG , Dolton G , et al. Metabolic adaptation of human CD4+ and CD8+ T‐cells to T‐cell receptor‐mediated stimulation. Front Immunol. 2017;8:1516. 29170670 10.3389/fimmu.2017.01516 PMC5684100

[12] van der Windt GJ , O'Sullivan D , Everts B , et al. CD8 memory T cells have a bioenergetic advantage that underlies their rapid recall ability. PNAS. 2013;110(35):14336‐14341. 23940348 10.1073/pnas.1221740110 PMC3761631

[13] Gubser PM , Bantug GR , Razik L , et al. Rapid effector function of memory CD8(+) T cells requires an immediate‐early glycolytic switch. Nat Immunol. 2013;14(10):1064‐1072. 23955661 10.1038/ni.2687

[14] Alfirevic A , Gonzalez‐Galarza F , Bell C , et al. In silico analysis of HLA associations with drug‐induced liver injury: use of a HLA‐genotyped DNA archive from healthy volunteers. Genome Med. 2012;4(6):51. 22732016 10.1186/gm350 PMC3698530

[15] Faulkner L , Gibson A , Sullivan A , et al. Detection of primary T cell responses to drugs and chemicals in HLA‐typed volunteers: implications for the prediction of drug immunogenicity. Toxicol Sci. 2016;154(2):416‐429. 27637899 10.1093/toxsci/kfw177

[16] Mauri‐Hellweg D , Bettens F , Mauri D , Brander C , Hunziker T , Pichler WJ . Activation of drug‐specific CD4+ and CD8+ T cells in individuals allergic to sulfonamides, phenytoin, and carbamazepine. J Immunol. 1995;155(1):462‐472. 7602118

[17] Mookerjee SA , Gerencser AA , Nicholls DG , Brand MD . Quantifying intracellular rates of glycolytic and oxidative ATP production and consumption using extracellular flux measurements. J Biol Chem. 2017;292(17):7189‐7207. 28270511 10.1074/jbc.M116.774471 PMC5409486

[18] Littlehales E , Murray O , Dunsmuir R . Vancomycin‐induced DRESS syndrome: an important concern in orthopedic surgery. Case Rep Orthop. 2018;2018:1439073. 30034896 10.1155/2018/1439073 PMC6035812

[19] Hewitson LJ . Vancomycin induced DRESS syndrome (drug reaction with eosinophilia and systemic symptoms) in a patient with tricuspid endocarditis. BMJ Case Rep. 2019;12:9. 10.1136/bcr-2019-229590 PMC6754717 31527200

[20] Cao Y , Rathmell JC , Macintyre AN . Metabolic reprogramming towards aerobic glycolysis correlates with greater proliferative ability and resistance to metabolic inhibition in CD8 versus CD4 T cells. PloS One. 2014;9(8):e104104. 25090630 10.1371/journal.pone.0104104 PMC4121309

[21] Frauwirth KA , Riley JL , Harris MH , et al. The CD28 signaling pathway regulates glucose metabolism. Immunity. 2002;16(6):769‐777. 12121659 10.1016/s1074-7613(02)00323-0

[22] Bauer DE , Harris MH , Plas DR , et al. Cytokine stimulation of aerobic glycolysis in hematopoietic cells exceeds proliferative demand. FASEB J. 2004;18(11):1303‐1305. 15180958 10.1096/fj.03-1001fje PMC4458073

[23] Gardner J , Ogese M , Betts CJ , Pirmohamed M , Naisbitt DJ . Characterization of Teicoplanin‐specific T‐cells from drug Naïve donors expressing HLA‐A*32:01. Chem Res Toxicol. 2022;35(2):199‐202. 35107993 10.1021/acs.chemrestox.1c00425 PMC9007560

[24] Ramadan G . Epstein‐Barr virus‐transformed B‐cells as efficient antigen presenting cells to propagate aspergillus‐specific cytotoxic T‐lymphocytes. Egypt J Immunol. 2008;15(1):145‐157. 20306679

[25] Ceuppens JL , Baroja ML , Lorre K , Van Damme J , Billiau A . Human T cell activation with phytohemagglutinin. The function of IL‐6 as an accessory signal. J Immunol. 1988;141(11):3868‐3874. 3263438

[26] Li Y , Kurlander RJ . Comparison of anti‐CD3 and anti‐CD28‐coated beads with soluble anti‐CD3 for expanding human T cells: differing impact on CD8 T cell phenotype and responsiveness to restimulation. J Transl Med. 2010;8(1):104. 20977748 10.1186/1479-5876-8-104 PMC2987859

[27] Trickett A , Kwan YL . T cell stimulation and expansion using anti‐CD3/CD28 beads. J Immunol Methods. 2003;275(1–2):251‐255. 12667688 10.1016/s0022-1759(03)00010-3

[28] Boise LH , Minn AJ , Noel PJ , et al. CD28 costimulation can promote T cell survival by enhancing the expression of Bcl‐XL. Immunity. 1995;3(1):87‐98. 7621080 10.1016/1074-7613(95)90161-2

[29] van der Windt GJW , Everts B , Chang CH , et al. Mitochondrial respiratory capacity is a critical regulator of CD8(+) T cell memory development. Immunity. 2012;36(1):68‐78. 22206904 10.1016/j.immuni.2011.12.007 PMC3269311

[30] Peter JG , Lehloenya R , Dlamini S , et al. Severe delayed cutaneous and systemic reactions to drugs: a global perspective on the science and art of current practice. J Allergy Clin Immunol Pract. 2017;5(3):547‐563. 28483310 10.1016/j.jaip.2017.01.025 PMC5424615

[31] Zhao Q , Alhilali K , Alzahrani A , et al. Dapsone‐ and nitroso dapsone‐specific activation of T cells from hypersensitive patients expressing the risk allele HLA‐B*13:01. Allergy. 2019;74(8):1533‐1548. 30844087 10.1111/all.13769 PMC6767778

[32] Monshi MM , Faulkner L , Gibson A , et al. Human leukocyte antigen (HLA)‐B*57:01‐restricted activation of drug‐specific T cells provides the immunological basis for flucloxacillin‐induced liver injury. Hepatol (Baltimore, Md). 2013;57(2):727‐739. 10.1002/hep.26077 22987284

[33] Bell CC , Faulkner L , Martinsson K , et al. T‐cells from HLA‐B*57:01+ human subjects are activated with abacavir through two independent pathways and induce cell death by multiple mechanisms. Chem Res Toxicol. 2013;26(5):759‐766. 23541086 10.1021/tx400060p

[34] Liu H , Rhodes M , Wiest DL , Vignali DAA . On the dynamics of TCR:CD3 complex cell surface expression and Downmodulation. Immunity. 2000;13(5):665‐675. 11114379 10.1016/s1074-7613(00)00066-2

[35] Boyer C , Auphan N , Luton F , et al. T cell receptor/CD3 complex internalization following activation of a cytolytic T cell clone: evidence for a protein kinase C‐independent staurosporine‐sensitive step. Eur J Immunol. 1991;21(7):1623‐1634. 1829410 10.1002/eji.1830210707

[36] MacDonald HR . Energy metabolism and T‐cell‐mediated cytolysis. II. Selective inhibition of cytolysis by 2‐deoxy‐D‐glucose. J Exp Med. 1977;146(3):710‐719. 302305 10.1084/jem.146.3.710 PMC2180795

[37] MacDonald HR , Cerottini JC . Inhibition of T cell‐mediated cytolysis by 2‐deoxy‐D‐glucose:dissociation of the inhibitory effect from glycoprotein synthesis. Eur J Immunol. 1979;9(6):466‐470. 315316 10.1002/eji.1830090610

[38] Nguyen H , Haarberg KM , Wu Y , et al. Allogeneic T cells utilize glycolysis As the predominant metabolic pathway to induce acute graft‐versus‐host disease. Blood. 2014;124(21):2419.

[39] Assmann JC , Farthing DE , Saito K , et al. Glycolytic metabolism of pathogenic T cells enables early detection of GVHD by 13C‐MRI. Blood. 2021;137(1):126‐137. 32785680 10.1182/blood.2020005770 PMC7808015

