# Supplementary material for: Glycolysis: An early marker for vancomycin‐specific T‐cell activation
Source: Clin Exp Allergy. 2024 Jan 4;54(1):21–33. doi: 10.1111/cea.14423 (PMC10953384; doi:10.1111/cea.14423)
Supplement: Supplementary file 1 — Figure S1 [file CEA-54-21-s001.pdf]

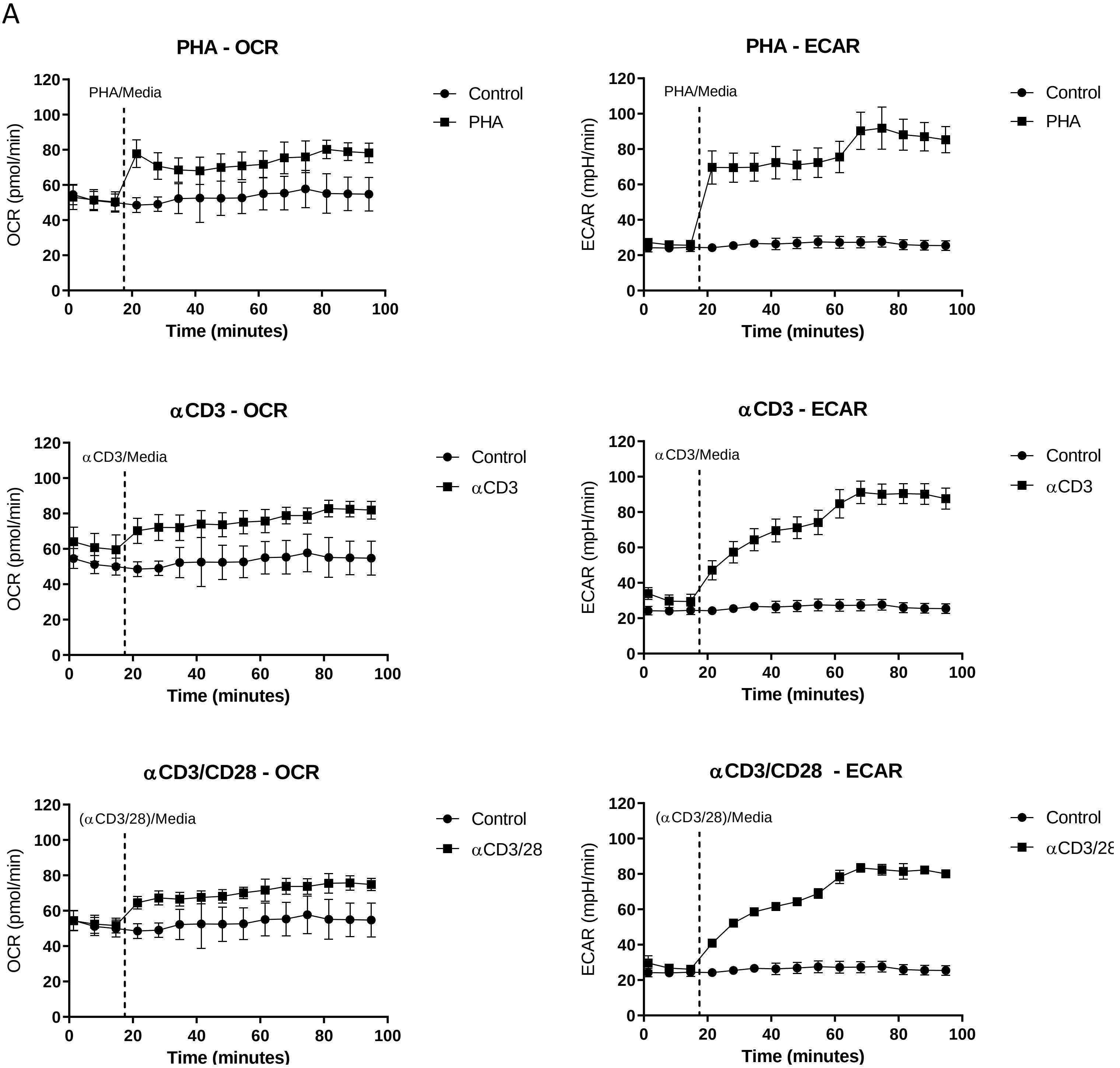

Supplementary Figure 1. Glycolytic dependence of vancomycin-specific TCCs after activation with PHA, αCD3 and αCD3/CD28 antibodies. (A) OCR and ECAR measurements of TCCs after acute injection of model stimulants. Drug-specific TCCs ($3x10^5$) were exposed to either PHA (10 µg/mL), αCD3 (10 µg/mL), αCD3/CD28 (10 µg/mL and 20 µg/mL) or Seahorse XF base medium only, via acute stimulation following the completion of 3 basal measurements in the absence of glucose. OCR (pmol/min) and ECAR (mpH/min) readouts were recorded by the Seahorse XFe96 Analyzer over a 96 min period.

Supplementary Figure 1

A

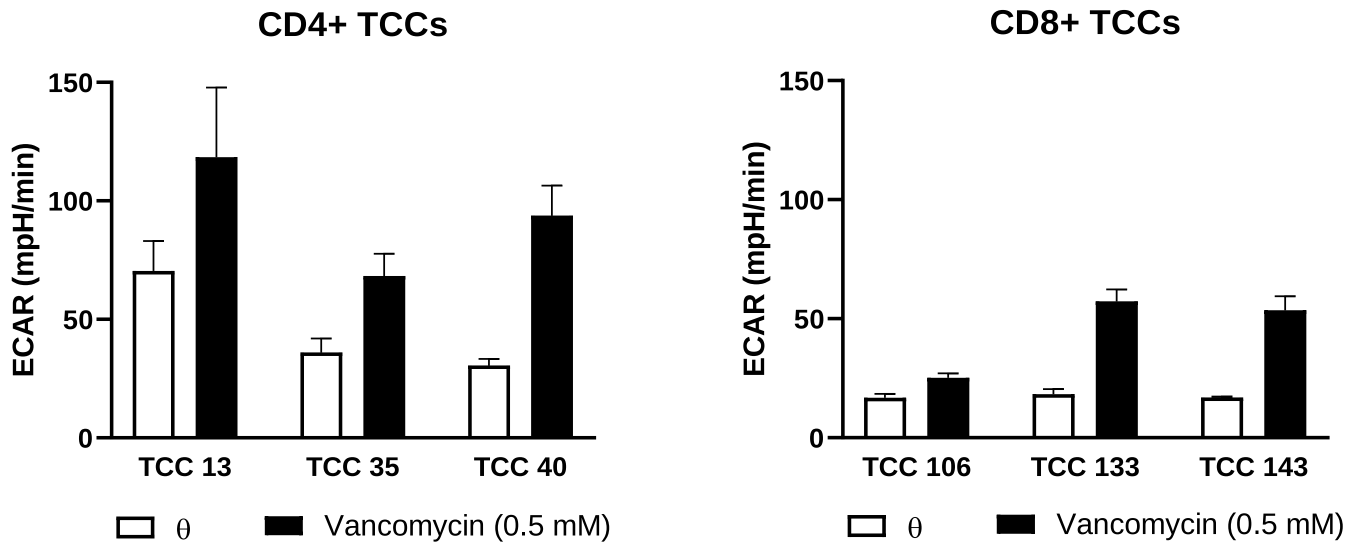

Supplementary Figure 2. Glycolytic dependence of CD4+ TCCs generated from a hypersensitive patient and CD8+ TCCs generated from a healthy donor following acute vancomycin exposure. (A) Maximal ECAR measurements of drug-reactive CD4+ and CD8+ TCCs after acute stimulation with vancomycin. $3x10^5$ CD4+ (n=3) and CD8+ TCCs (n=3) were sequentially exposed to D-glucose (25 mM), APCs ($5x10^4$) and vancomycin (0.5 mM) followed by a final injection of 2-DG (25 mM). Maximal ECAR values (mpH/min) were measured relative to basal ECAR levels of control TCCs.

Supplementary Figure 2

A

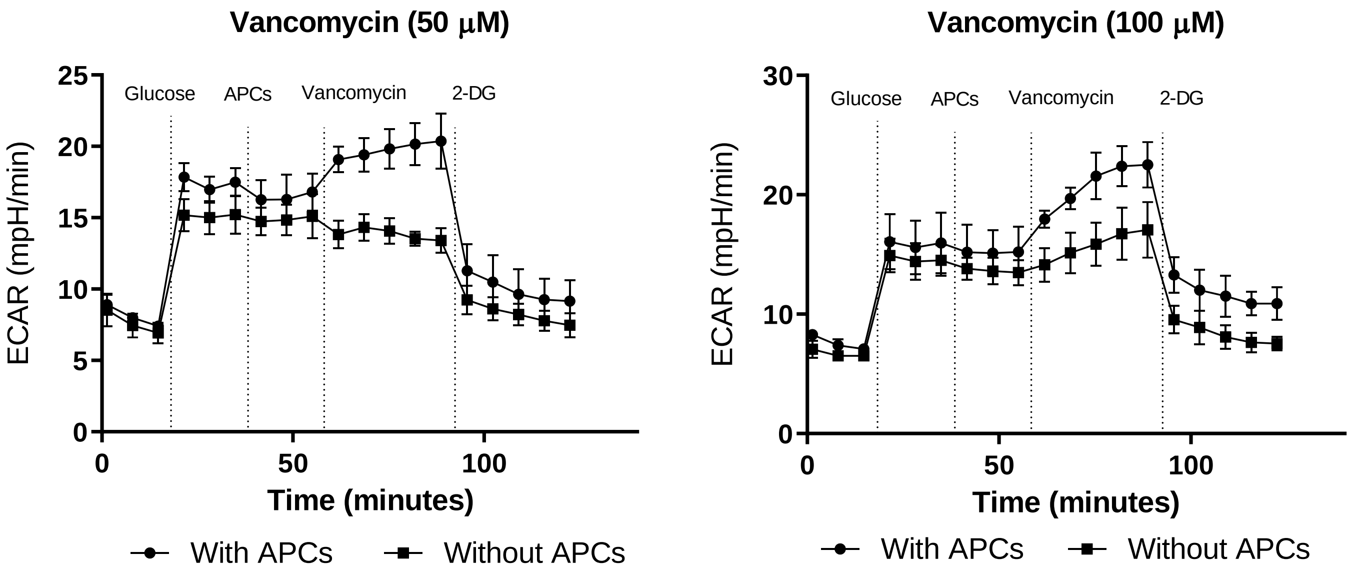

Supplementary Figure 3. Glycolysis Stress Test of CD8+ vancomycin-specific TCCs assessing energetic response to vancomycin with and without APC exposure. (A) Glycolysis Stress Test to determine APC functionality. Vancomycin-specific TCCs were injected with D-glucose (25 mM), APCs ($5x10^4$) or Seahorse XF base medium only. Following injection with D-glucose +/- EBV-transformed B-cells, cultures were exposed to low, titrated concentrations of vancomycin (50 µM and 100 µM) followed by a final injection of 2-DG (25 mM).

Supplementary Figure 3
