# Supplementary material for: Glycolysis: An early marker for vancomycin‐specific T‐cell activation
Source: Clin Exp Allergy. 2024 Jan 4;54(1):21–33. doi: 10.1111/cea.14423 (PMC10953384; doi:10.1111/cea.14423)
Supplement: Supplementary file 2 — Appendix S1 [file CEA-54-21-s002.docx]

### Supplementary Text – Methods

### Generation of vancomycin-specific T-cell clones

PBMC were seeded at 1x10^6^ PBMC/well with vancomycin and incubated for a 14-day period (37 °C, 5% CO_2_). On days 6 and 9, PBMC cultures were supplemented with fresh Roswell Park Memorial Institute (RPMI) medium containing 10% human AB serum, HEPES (25 mM), penicillin (1000 U/mL), streptomycin (0.1 mg/mL), L-glutamine (2 mM) and transferrin (25 µg/mL). RPMI medium was further supplemented with IL-2 (200 U/mL) in order to promote and facilitate the expansion drug-specific T-cells. On day 14 T-cells were enriched for either CD4+ or CD8+ expressing populations by MACS separation with the use of CD8+ microbeads and clones were generated using a serial dilution protocol [1]. Briefly, irradiated allogenic PBMC (5x10^5^ cells/mL), T-cells, IL-2 (200 U/mL) and PHA (5 µg/mL) were transferred to 96-well U-bottomed plates to achieve an average of 1 T-cell/well. Cultures were incubated for 5 days (37 °C, 5% CO_2_) and maintained with fresh medium supplemented with IL-2 (200 U/mL) every 2 days thereafter for 14 days.

After 14 days, cultures were further restimulated with freshly isolated allogenic PBMC (5x10^5^ cells/mL) diluted in medium supplemented with IL-2 (400 U/mL), and PHA (10 µg/mL). Cultures were then maintained every 2 days as previously described and plates were visually inspected for clonal expansion. Confluent TCCs were split into 4 wells and maintained twice per week prior to specificity testing during which TCCs were rechallanged with RPMI medium or vancomycin alongside irradiated autologous Epstein-Barr virus (EBV) transformed B-cells. Test cultures were incubated for 48 h (37 °C, 5% CO_2_) and pulsed with tritiated [^3^H]-thymidine (0.5 µCi/well) for the final 16 h of incubation to allow incorporation into cellular DNA before scintillation counting.

**Dose-response analysis of TCCs**

Once clones were deemed to show specificity during initial testing and sufficient mitogen driven expansion of clonal populations had taken place, it was necessary to confirm these TCCs as drug-responsive at graded concentrations of vancomycin. Drug-specific TCCs were washed in medium, to exclude IL-2, and resuspended at 5x10^5^ cells/mL. Autologous EBV-transformed B-cells were irradiated and resuspended in PPMI medium, at 2x10^5^ cells/mL. Test cultures were set up within a 96-well U-bottomed plate in triplicate to allow for statistical analysis. Each condition consisted of TCCs (5x10^4^ cells), EBV-transformed B-cells (1x10^4^ cells) and graded concentrations of drug added in 50 µL aliquots, 4x the desired final concentration to assess dose dependency. PHA (10 µg/mL) was included to verify the viability and proliferative potential of cultures (positive control), with the addition of RPMI medium in isolation providing an indication of basal proliferative levels. Drug-treated cultures were incubated for 48 h (37 °C, 5% CO_2_) and subjected to [^3^H]-thymidine incorporation (0.5 µCi/well) as previously described.

**Phenotypic analysis of vancomycin-specific TCCs**

Phenotyping of T-cells to determine CD4+/CD8+ expression was performed using fluorophore conjugated CD4/CD8 antibodies (BD Biosciences), and experiments were conducted according to the manufacturer’s instructions. Briefly, 50-100 µL of cell suspension, confluency depending, were stained with conjugated fluorophores (FITC, 3 µL; APC, 3 µL; PE, 0.5 µL) for 20 mins at 4 °C. Following incubation, stained T-cells were washed with 2 mL FACS buffer and centrifuged at 1500 rpm for 10 min at 4 °C. Supernatant was discarded and cells were then resuspended in 100-200 µL 4% paraformaldehyde (PFA) solution. TCCs were analysed for 10^4^ events using a FACS-Canto II instrument.

**Cytokine release assays (ELISpot)**

Antigen-specific cytokine release from drug-specific TCCs was assessed using an enzyme-linked immunospot (ELISpot) assay. TCCs (5x10^4^ cells) were co-cultured in a 96-well U-bottomed plate with irradiated autologous EBV-transformed B-cells (1x10^4^ cells) and study compounds. Drug exposed cultures were incubated for 24 h (37 °C, 5% CO_2_). On the same day, Protein Binding Immobilon-P Membrane 96-well Multiscreen® filter plates were activated by the addition of 35% ethanol solution (60 secs) before being thoroughly washed (5x) with 200 µL distilled water. Activated Multiscreen® filter plates were then coated with 100 µL of capture antibody (IFN-y) diluted in sterile HBSS according to the manufacturer’s instructions (Mabtech, Nacka Strand, Sweden). Coated plates were then incubated for 24 h at 4 °C.

The next day, ELISpot plates pre-coated for cytokines of interest were washed thoroughly (5x) with 200 µL HBSS to remove any remaining capture antibodies, before being blocked at RT with 200 µL RPMI medium containing 10% human AB serum for 2 h. After blocking, co-cultures of T-cells, EBV-transformed B-cells and drug were transferred (200 µL volume) into corresponding wells of the pre-coated ELISpot plate and incubated for a further 24 h (37 °C, 5% CO_2_). On day 2, cells were discarded and ELISpot plates were washed (5x) with 200 µL HBSS. Detection antibodies, conjugated with biotin, corresponding to the cytokines of interest were diluted in HBSS supplemented with 0.5% FBS. 100 µL of biotinylated antibody solution was added to each well of the assay plate and incubated for 2 h at RT. Following incubation, plates were washed (5x) with 200 µL HBSS and 100 µL of streptavidin conjugated alkaline phosphatase (strep-ALP) was added to each well and plates were incubated for 1 hr at RT. Sterile filtered (0.45 µM) BCIP-NBT substrate solution was added to each assay well following the removal of strep-ALP and further washing of the plate (5x; HBSS). During the development process, plates were incubated at RT for 15-20 mins in the dark to allow colorimetric reaction to occur. Following assay completion, cessation of the reaction was achieved by washing plates with an excess of cold water. Developed ELISpot plates were dried overnight and spots were imaged and quantified the following day using an AID ELISpot reader (Cadima Madical, Stourbridge, UK).

**MHC blocking assays**

Major histocompatibility complex (MHC) blocking antibodies were used to assess the dependence of drug-specific TCCs for specific human leukocyte antigen (HLA) complexes and can help elucidate HLA restricted T-cell activation by proliferative blockade. Drug-specific TCCs (5x10^4^ cells) were co-cultured in a 96-well U-bottomed plate with irradiated autologous EBV-transformed B-cells (1x10^4^ cells). Cultures were then incubated with HLA blocking antibodies (HLA-ABC, HLA-DR, DP, DQ, HLA-DR; 10 µg/mL) prior to drug exposure for 1 h to pre-emptively block T-cell self-presentation. After 1 h, RPMI medium (negative control) or soluble drug was added to the co-culture and cells were incubated for 48 h (37 °C, 5% CO_2_). Cultures were then pulsed with tritiated [^3^H]-thymidine (0.5 µCi/well) for an additional 16 h and proliferation after HLA blockade was determined by scintillation counting as previously described.

**Glycolysis Stress Test**

Novel methods were developed to assess energetic phenotypes of single T-cell populations after acute stimulation. Glycolysis-stress based assays were optimised for target cell adherence, APC incorporation, use of glycolytic inhibitors (2-DG) and drug/stimulant injection.

One day prior to assay commencement, Seahorse XFe96 cell culture microplates were coated with 25 µL Corning® Cell-Tak™ cell and tissue adhesive solution as a concentration of 22.4 µg/mL, diluted in sterile filtered NaHCO_3_ (pH 8.0). Coated microplates were incubated at RT for 1 h before being washed with (x2) with sterile water and stored at 4 °C overnight. On the same day, the Seahorse xFe96 sensor cartridge was hydrated with 200 μL Seahorse XF calibrant solution and incubated overnight at 37 °C. Autologous EBV-transformed B-cells for acute injection within the assay were irradiated for 20 min, resuspended in RPMI medium and incubated overnight (37 °C, 5% CO_2_).

On the day of the assay, the pH of Seahorse XF base medium was adjusted to 7.4 and the coated cell culture microplate was brought up to RT, prior to cellular addition. Seahorse XF base medium (pH 7.4) was supplemented with L-glutamine (2 mM) and drug-specific TCCs were harvested from culture plates, counted and resuspended in Seahorse XF base medium. Cells were then transferred into coated Seahorse XFe96 cell culture microplates in 50 µL aliquots (3x10^5^/cells/well) and centrifuged at 1500 rpm for 2 mins to facilitate adherence. Cell culture microplates containing adhered T-cell cultures were then incubated in the absence of CO_2_ for 1 h at 37 °C. Irradiated EBV-transformed B-cells were counted, centrifuged and diluted in Seahorse XF base medium to 2x10^6^/mL. Injection ports within the Seahorse XFe96 sensor cartridge were loaded with 25 mM D-glucose (Port A), 5x10^4^ EBV-transformed B-cells (Port B), the study compound (Port C) and 25 mM 2-DG (Port D) in 25 µL aliquots. Following successful calibration, the assay plate was inserted into the Seahorse XFe96 Analyzer and energetic readouts, specifically the oxygen consumption rate (OCR) and extracellular acidification rate (ECAR), were measured in TCCs after acute drug exposure.

**CD3 downregulation assays**

To explore the time points at which markers of drug-specific T-cell activation are detectable, extracellular CD3 expression was studied as a biomarker for TCR triggering. Co-cultures of TCCs (5x10^4^ cells/well) and autologous EBV-transformed B-cells (1x10^5^ cells/well) were pulsed with drug for multiple time points (48 h, 24 h, 4 h, 2 h, 1 h, 30 min, 10 min, 5 min) in 96-well U-bottomed plates. Following incubation of the final time point, the drug exposed cell suspensions were transferred to FACS tubes and stained with 5 μL anti-CD3 antibody for 15 mins 4 °C. Stained cells were washed in FACS buffer to remove unbound antibody and resuspended in 4% PFA and stored at 4 °C. Both unstained and untreated cultures were set up in parallel for comparison and samples were analysed for 10^4^ events using a FACS-Canto II instrument. Reduced CD3 (APC) fluorescence intensity, was indicative of downregulation and drug-specific T-cell activation.

**Reference**

1. Mauri-Hellweg, D., et al., *Activation of drug-specific CD4+ and CD8+ T cells in individuals allergic to sulfonamides, phenytoin, and carbamazepine.* J Immunol, 1995. **155**(1): p. 462-72.
